# CXCL13-producing CD4^+^ T cells accumulate in the early phase of tertiary lymphoid structures in ovarian cancer

**DOI:** 10.1172/jci.insight.157215

**Published:** 2022-06-22

**Authors:** Masayo Ukita, Junzo Hamanishi, Hiroyuki Yoshitomi, Koji Yamanoi, Shiro Takamatsu, Akihiko Ueda, Haruka Suzuki, Yuko Hosoe, Yoko Furutake, Mana Taki, Kaoru Abiko, Ken Yamaguchi, Hidekatsu Nakai, Tsukasa Baba, Noriomi Matsumura, Akihiko Yoshizawa, Hideki Ueno, Masaki Mandai

**Affiliations:** 1 Department of Gynecology and Obstetrics and; 2 Department of Immunology, Kyoto University Graduate School of Medicine, Kyoto, Japan.; 3 Institute for the Advanced Study of Human Biology (ASHBi), Kyoto University, Kyoto, Japan.; 4 Department of Obstetrics and Gynecology, National Hospital Organization Kyoto Medical Center, Kyoto, Japan.; 5 Department of Obstetrics and Gynecology, Kindai University Faculty of Medicine, Osaka, Japan.; 6 Department of Obstetrics and Gynecology, Iwate Medical University, Iwate, Japan.; 7 Department of Diagnostic Pathology, Kyoto University Graduate School of Medicine, Kyoto, Japan.

**Keywords:** Immunology, Oncology, Adaptive immunity, Cancer, Obstetrics/gynecology

## Abstract

Tertiary lymphoid structures (TLS) are transient ectopic lymphoid aggregates whose formation might be caused by chronic inflammation states, such as cancer. However, how TLS are induced in the tumor microenvironment (TME) and how they affect patient survival are not well understood. We investigated TLS distribution in relation to tumor infiltrating lymphocytes (TILs) and related gene expression in high-grade serous ovarian cancer (HGSC) specimens. CXCL13 gene expression correlated with TLS presence and the infiltration of T cells and B cells, and it was a favorable prognostic factor for patients with HGSC. Coexistence of CD8^+^ T cells and B cell lineages in the TME significantly improved the prognosis of HGSC and was correlated with the presence of TLS. CXCL13 expression was predominantly coincident with CD4^+^ T cells in TLS and CD8^+^ T cells in TILs, and it shifted from CD4^+^ T cells to CD21^+^ follicular DCs as TLS matured. In a mouse ovarian cancer model, recombinant CXCL13 induced TLS and enhanced survival by the infiltration of CD8^+^ T cells. These results suggest that TLS formation was associated with CXCL13-producing CD4^+^ T cells and that TLS facilitated the coordinated antitumor response of cellular and humoral immunity in ovarian cancer.

## Graphical abstract

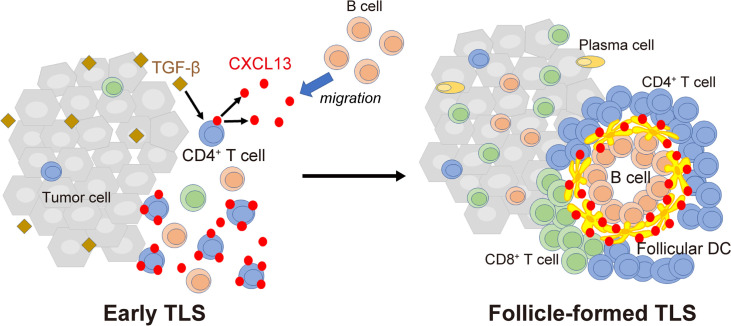

## Introduction

It is generally considered that the generation and regulation of an efficient adaptive immune response to cancer occurs in secondary lymphoid organs (SLOs), such as the regional lymph nodes. Antitumor immune cells are educated to recognize tumor antigens, proliferate in regional lymph nodes away from the tumor site, and then migrate into the tumor microenvironment (TME) to exert antitumor activity (1). Clinical studies have shown that higher densities of T cell subsets within the TME are associated with improved patient survival in several cancers, including ovarian cancer (2–5). We previously reported that the forced infiltration of CD8^+^ T cells into the TME by CCL19, a chemokine that attracts T cells, suppressed tumors in an ovarian cancer model (6). These results indicate that T cells have a critical role in the TME and that the TME might be a therapeutic target if effectively altered by immune-activating signals such as chemokines. To date, strategies to enhance the clinical efficacy of antitumor treatments have predominantly focused on the T cell component in the tumor, and the roles of other immune cell components have not been fully elucidated.

Recent studies have revealed an alternative immune response at the tumor site within SLO-like cellular aggregates called tertiary lymphoid structures (TLS) (7). TLS are transient ectopic lymphoid aggregates whose formation might be caused by chronic inflammation states, including autoimmune and infectious diseases, transplanted organ rejection, and cancer (7–9). The presence of TLS is associated with a favorable prognosis in most solid malignancies (10). Recently, it was reported that TLS-associated B cells synergized with T cells to contribute antitumor effects and that the presence of TLS and B cells in tumor sites enhanced the efficacy of immunotherapy (11–14). The recognition of the relevance of TLS to cancer has led to a growing interest in TLS as an immunomodulatory target to enhance tumor immunity, although how this can be induced therapeutically is not known.

The chemokines CXCL13, CCL19, and CCL21 are involved in lymphoid tissue inducer (LTi) cell homing and lymph node development (8). CXCL13 was reported to be essential for the initial attraction of LTi cells and the formation of early lymph nodes (15). Although TLS are thought to share the mechanisms of initial development with SLOs, TLS formation is distinct from the preprogrammed processes involved in SLOs and does not necessarily occur in all patients. The generation of TLS in inflamed tissues might be governed by specific inflammatory signals that have not been fully identified (7). In autoimmune diseases such as rheumatoid arthritis (RA), we showed that TGF-β and other proinflammatory cytokines enhanced CXCL13 production by CD4^+^ T cells and that CXCL13-producing CD4^+^ T cells had an important role in the formation of TLS (16, 17). However, whether the same mechanism can be applied to the case of malignant tumors including ovarian cancer has not been investigated.

In this study, we assessed the relationship between tumor infiltrating T or B cells subsets and the presence of TLS, and we evaluated the prognostic impact of TLS in ovarian cancer. We also investigated whether CXCL13 was associated with TLS formation and improved the prognosis of patients with ovarian cancer. The role of CXCL13-producing CD4^+^ T cells in the generation of TLS was also investigated.

## Results

### Intratumor infiltration of T cells and B cell lineages improves the prognosis of ovarian cancer and is associated with the presence of TLS.

**Figure 1:**
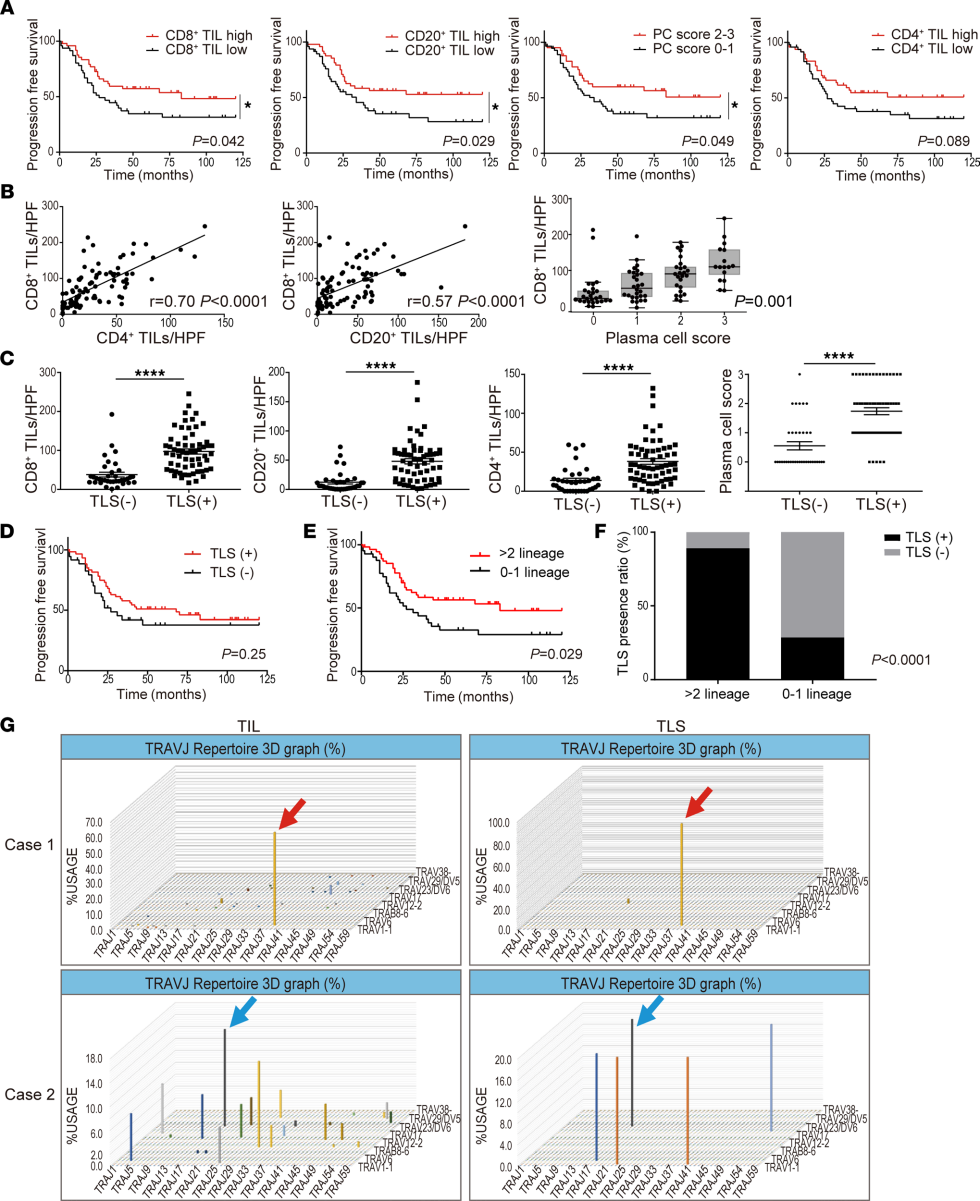
Intratumor infiltration of T cells and B cell lineages improves the prognosis of ovarian cancer and is associated with the presence of TLS. (**A**) Progression-free survival of the cohort stratified by respective infiltration of T and B cell subsets (*n* = 97, each). (**B**) Association between infiltrated numbers of CD4^+^ T cells and CD8^+^ T cells, and CD8^+^ T cells and B cell lineage s in tumors (*n* = 97). Correlations were determined by Pearson’s correlation test and Jonckheere-Terpstratrend tests. The *y* axis represent the mean count of 5 HPFs. (**C**) Characterization of the immune infiltrate in tumors according to TLS presence (TLS^−^, *n* = 36; TLS^+^, *n* = 61). *P* values were determined by Mann-Whitney *U* test. (**D**) Progression-free survival of patients with HGSC related to the presence of TLS. (**E**) Progression-free survival based on lymphocyte infiltration pattern (>2 lineages, *n* = 55; 0–1 lineage, *n* = 42). Analyses were performed with Kaplan-Meier estimates and log-rank tests in **A**, **D**, and **E**. (**F**) TLS presence ratio based on lymphocyte infiltration patterns. Analysis was performed by Fisher’s exact test. (**G**) TCR repertoire analysis separating TLS and TIL regions. The horizontal axis represents the J gene, the depth represents the V gene, and the vertical axis represents the frequency of usage. Clones indicated by arrows of the same color confirm the same amino acid sequence of CDR3.

The prognostic significance of the respective infiltration of T cell and B cell subsets was investigated by the IHC analysis of initial surgical specimens of high-grade serous ovarian cancer (HGSC) (*n* = 97) (Supplemental Table 1; supplemental material available online with this article; https://doi.org/10.1172/jci.insight.157215DS1). Patients with a higher infiltration of CD8^+^ T cells, CD20^+^ B cells, and CD38^+^ plasma cells (PC score 2–3) had significantly prolonged progression-free survival (PFS) than those with low numbers of infiltrating cells (*P* < 0.05, each) (Figure 1A). In addition, there was a significant correlation between the infiltrated numbers of CD8^+^ T cells and CD4^+^ T cells, and between the infiltrated numbers of CD8^+^ T cells and B cell lineage cells (Figure 1B).

H&E and IHC staining evaluation of TLS in the same samples (*n* = 97) revealed that 61 patients (62.9%) had TLS (Supplemental Table 1 and Supplemental Figure 1, A and B). In the cases with TLS, the number of infiltrating CD8^+^ T cells, CD4^+^ T cells, CD20^+^ B cells, or CD38^+^ plasma cells (plasma cell score) was significantly higher than in those without TLS (*P* < 0.0001, each) (Figure 1C). However, PFS did not differ with or without the presence of TLS (*P* = 0.25) (Figure 1D). To examine how TLS were related to the improved prognosis, we focused on the pattern of tumor infiltrating lymphocytes (TIL) infiltration and the presence of TLS. The presence of more than 2 of any lineages of infiltrating lymphocytes yielded statistical significance related to the PFS (*P* = 0.029) (Figure 1E and Supplemental Figure 1C). The presence of TLS was significantly associated with the intratumoral infiltration of more than 2 lineages of lymphocytes (*P* < 0.0001; Figure 1F and Supplemental Figure 1D).

The close relationship between TLS and the infiltrative distribution of various types of lymphocytes suggests that TLS might support TIL infiltration and proliferation. Next, we performed T cell receptor (TCR) and B cell receptor (BCR) repertoire analysis using tumor sections from patients with HGSC (*n* = 3), separating TLS and TIL regions by macrodissection (Supplemental Figure 2A). In 2 of 3 cases, TCR repertoire analysis showed many clones distributed in the TIL, whereas oligoclonal amplification was observed in TLS. Furthermore, the clone with the highest amplification from TIL was consistent with the clone observed in TLS (Figure 1G), suggesting that antigen-specific T cells that proliferated in TLS might also infiltrate into the tumor as TIL. The same situation was not observed in the TCR repertoire analysis of the remaining case and in the BCR repertoire analysis of 3 cases (Supplemental Figure 2B).

### CXCL13 gene expression in tumors is significantly correlated with the presence of TLS, lymphocyte infiltration, and a favorable prognosis for ovarian cancer.

**Figure 2:**
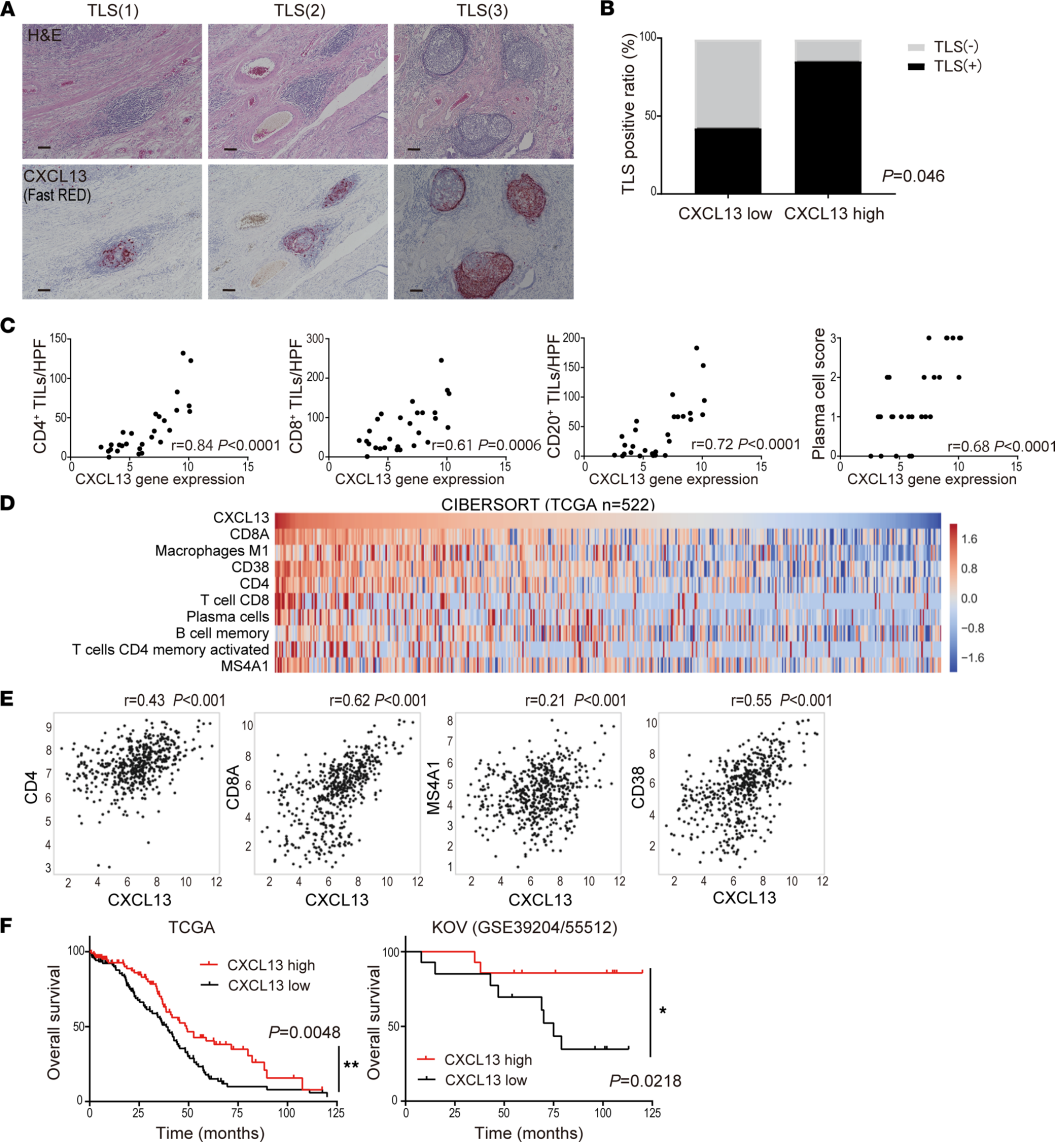
CXCL13 gene expression in tumors is significantly correlated with the presence of TLS, lymphocyte infiltration, and a favorable prognosis for ovarian cancer. (**A**) Representative TLS in tissues stained by H&E and CXCL13 (Fast RED) by RNA ISH. Scale bars: 100 μm. (**B**) TLS presence ratio based on CXCL13 gene expression. Analysis by Fisher’s exact test in 28 cases with microarray data. (**C**) Characterization of the immune infiltrate in tumors according to CXCL13 gene expression (*n* = 28). Correlation was determined by Spearman’s correlation test. (**D**) The distribution of infiltrating immune cells into the tumor site and CXCL13 gene expression using CIBERSORT (*n* = 522). (**E**) Correlation of CXCL13 gene expression with CD4, CD8A, MS4A1, and CD38 in CIBERSORT. Correlation was determined by Spearman’s correlation test. (**F**) Overall survival of patients with HGSC by CXCL13 gene expression (TCGA, *n* = 217; KOV, *n* = 28). Patients with CXCL13^hi^ defined if CXCL13 gene expression was above the median. Analyses were performed with Kaplan-Meier estimates and log-rank tests. The level of significance was set as *P < 0.05 and **P < 0.01.

The expression of CXCL13 analyzed by RNA in situ hybridization (ISH) demonstrated that CXCL13 was highly expressed in TLS (Figure 2A). In addition, there were cases in which immune cells in the tumor stroma also expressed high levels of CXCL13 (Supplemental Figure 3A). From the IHC of initial surgical specimens with our original microarray data (KOV, GSE39204/55512, *n* = 28), the ratio of cases with TLS was significantly higher in those with high CXCL13 gene expression than in those with low CXCL13 gene expression in tumor specimens (*P* = 0.046; Figure 2B). The expressions of CCL19 and CCL21 — cytokines also involved in TLS formation — were not significantly associated with TLS presence (Supplemental Figure 4A). Additionally, CXCL13 gene expression was significantly correlated with the numbers of several types of TILs such as CD4^+^ and CD8^+^ T cells, CD20^+^ B cells, and CD38^+^ plasma cells (*P* < 0.001, each) (Figure 2C and Supplemental Figure 3B).

CIBERSORT using the microarray data of ovarian cancer cases registered in The Cancer Genome Atlas (TCGA) confirmed a wide range of CXCL13 expression in TME and that CXCL13 expression correlated with the infiltration of various TILs. The infiltration of CD8^+^ T cells had the strongest correlation with CXCL13 gene expression; the infiltration of CD4^+^ T cells, CD20^+^ B cells, CD38^+^ plasma cells, and M1-macrophages also showed a strong correlation with CXCL13 gene expression, while that of M2-macrophages and mature DCs were negatively correlated and that of NK cells and Tregs were not correlated with CXCL13 gene expression (Figure 2, D and E, and Supplemental Figure 3, C and D). These results suggest that CXCL13 gene expression strongly correlated with the formation of TLS and the number of tumor-infiltrating T cells and B cell lineages.

Next, we examined the impact of CXCL13 on the prognosis of HGSC and found that patients with high CXCL13 gene expression had a significantly better prognosis in the KOV data and TCGA data (*P*<0.05, each) (Figure 2F). The expressions of CCL19 and CCL21 correlated with the numbers of several types of infiltrating TILs, but they were not significantly associated with prognosis (Supplemental Figure 4, B and C).

### CXCL13 produced by CD4^+^ T cells is critical for TLS initiation.

**Figure 3:**
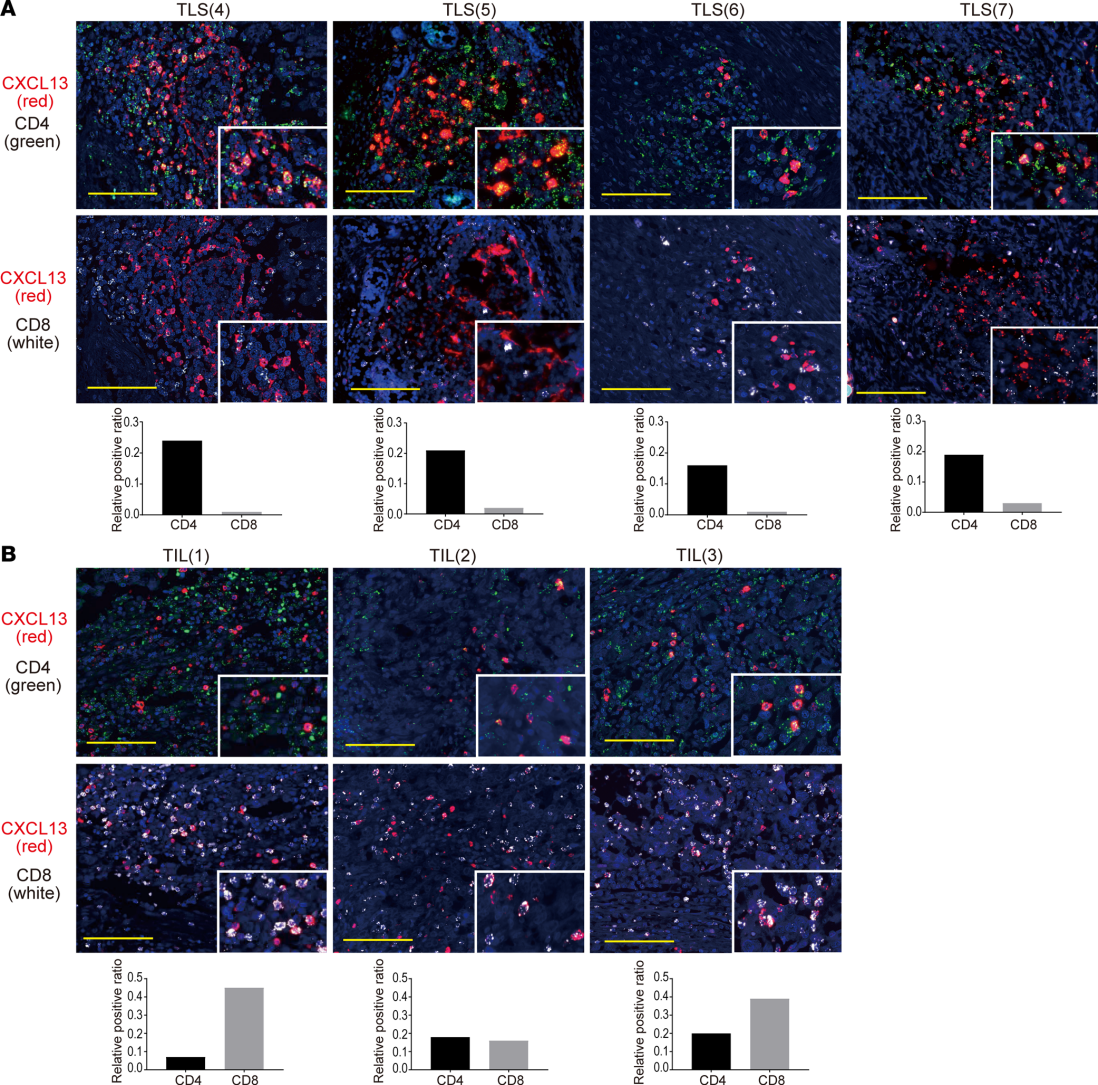
CXCL13 is mainly produced by CD4^+^ T cells in TLS. (**A**) Fluorescent double staining of CXCL13 (red) and CD4 (green), and CXCL13 (red) and CD8 (white) by RNA ISH in TLS. Images of 4 representative TLS are shown. (**B**) Fluorescent double staining of CXCL13 (red) and CD4 (green), and CXCL13 (red) and CD8 (white) by RNA ISH in TIL. The upper and lower pictures are representative TIL images from the same patient. Nuclei are stained with DAPI (blue). Scale bars: 100 μm. Colocalization of CXCL13 with CD4 or CD8 is shown in the bar graph as the relative positive ratio quantified using BZ-H4C/hybrid cell count software.

To identify cells producing CXCL13 involved in TLS formation, we performed RNA ISH double staining (CXCL13 and CD4^+^ T cells, CXCL13 and CD8^+^ T cells) using HGSC tumor specimens. In the TLS region, CXCL13 was highly coexpressed with CD4^+^ T cells, whereas CD8^+^ T cells predominantly expressed CXCL13 in the tumor and stromal regions (Figure 3, A and B).

**Figure 4:**
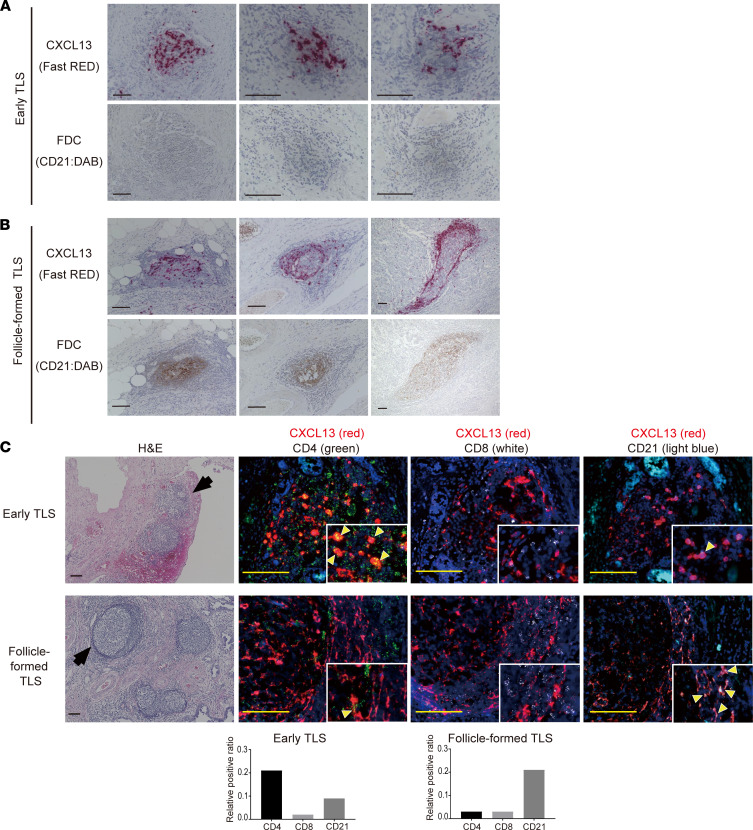
The source of CXCL13 production in TLS shifts to CD21^+^ FDC with the maturation of TLS. (**A**) Representative images of early TLS. Upper panels show CXCL13 (RNA ISH, Fast RED) and lower panels show FDC (CD21 IHC, DAB). (**B**) Representative images of follicle-formed TLS. The middle panel is the same case as TLS(2), found in Figure 2A, which shows one of the most typical Follicle-formed TLS. The presence of CD21^+^ FDC is shown in the bottom panel. (**C**) Fluorescence double staining of CXCL13 (red) and CD4 (green), CXCL13 (red) and CD8 (white), and CXCL13 (red) and CD21 (light blue) in representative early TLS and follicle-formed TLS. The same Early TLS shown in Figure 3A was used to show the cell source of CXCL13 expression. Nuclei are stained with DAPI (blue). Scale bar: 100 μm. Colocalization of CXCL13 with CD4, CD8, or CD21 is shown in the bar graph as the relative positive ratio quantified using BZ-H4C/hybrid cell count software.

HGSC tissues contain 2 types of TLS: early TLS, in which lymphocytes aggregate diffusely and CD21^+^ cells are scarce, and follicle-formed TLS, which has the follicular morphology of SLO and where CD21^+^ follicular DCs (FDCs) are distributed in a reticular pattern (Figure 4, A and B). Therefore, we performed the double staining of CXCL13 and CD8^+^ T cells, CXCL13 and CD4^+^ T cells, and CXCL13 and CD21^+^ FDCs by RNA ISH for representative early TLS and follicle-formed TLS. CXCL13 expression was highly consistent with CD4^+^ cells in early TLS, whereas few CD4^+^ T cells expressing CXCL13 were observed in follicle-formed TLS and CXCL13 expression was highly consistent with spindle-shaped CD21^+^ FDCs (Figure 4C). These results imply that CXCL13-producing CD4^+^ T cells are involved in the early stage of TLS formation.

### TGF-β promotes the production of CXCL13.

**Figure 5:**
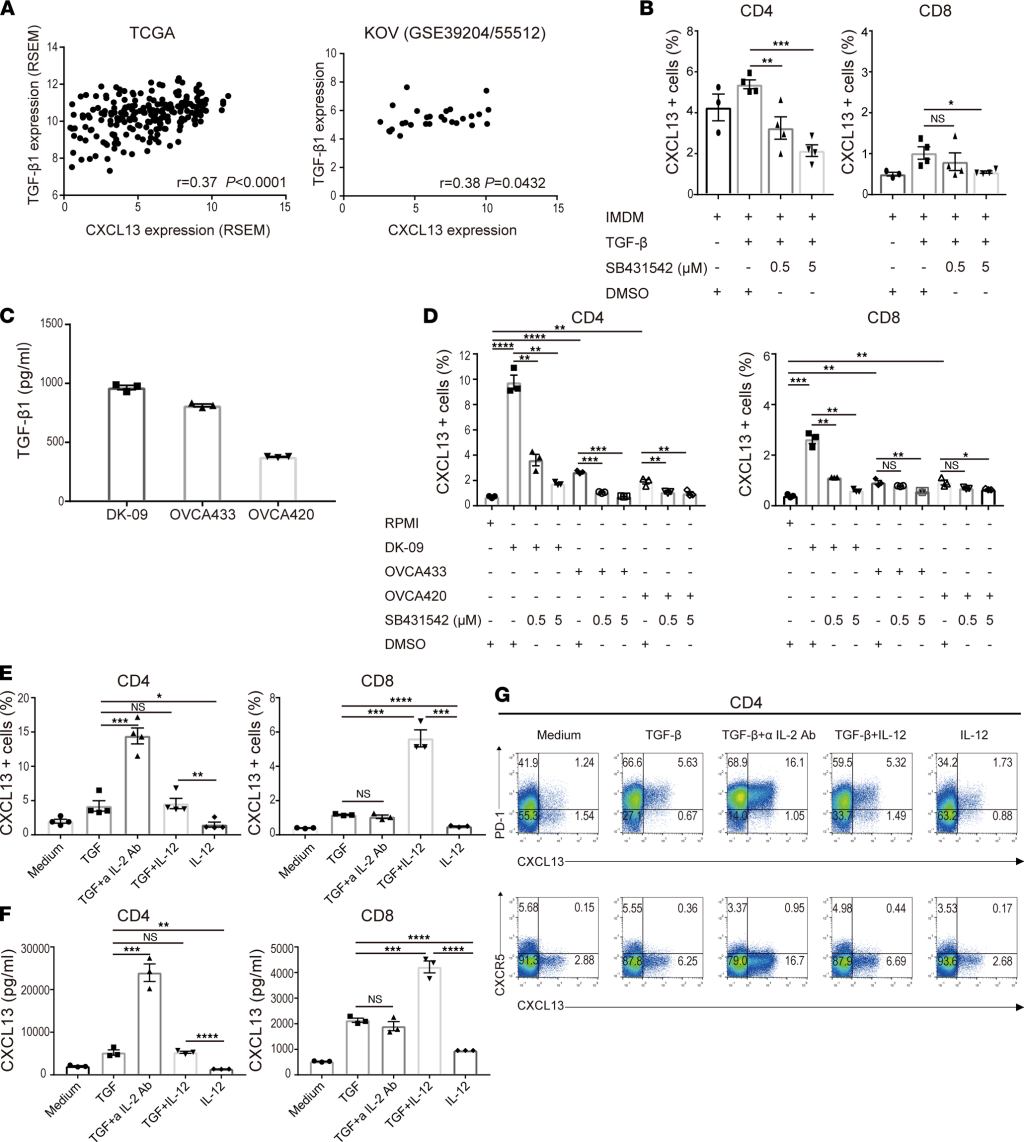
TGF-β promotes the production of CXCL13. (**A**) Correlation between CXCL13 and TGF-β1 expression in TCGA (*n* = 217) and KOV (*n* = 28). Correlation was determined by Spearman’s correlation test. (**B**) Human naive CD4^+^ and CD8^+^ T cells from a healthy donor were differentiated by TCR stimulation and TGF-β1 in the presence or absence of a TGF signal inhibitor, SB431542. The proportion of CXCL13^+^ cells was determined by flow cytometry. Data are shown as the mean ± SEM (*n* = 3–4). Statistical significance was determined by 2-tailed Student’s *t* test. *P < 0.05, **P < 0.01, and ***P < 0.001. (**C**) The concentration of TGF-β1 in conditioned medium obtained from 3 human ovarian cancer cell lines was measured by ELISA. Data are shown as the mean ± SEM (*n* = 3). (**D**) Human naive CD4^+^ and CD8^+^ T cells from a healthy donor were differentiated with TCR stimulation and conditioned medium obtained from 3 human ovarian cancer cell lines in the presence or absence of a TGF signal inhibitor, SB431542. The proportion of CXCL13^+^ cells was determined by flow cytometry. Data are shown as the mean ± SEM (*n* = 3). (**E**–**G**) Human naive CD4^+^ and CD8^+^ T cells from a healthy donor were differentiated with TCR stimulation and the indicated cytokines. The proportion of CXCL13^+^ cells was determined by flow cytometry (**E**). The concentration of CXCL13 in the culture supernatant was measured by ELISA (**F**). Data are shown as the mean ± SEM (*n* = 3–4). Statistical significance was determined by 2-tailed Student’s *t* test. *P < 0.05, **P < 0.01, ***P < 0.001, ****P < 0.0001. Representative dot plots of PD-1 (upper row), CXCR5 (lower row), and intracellular CXCL13 in healthy human naive CD4^+^ T cells are shown (**G**).

To investigate which factors promote CXCL13 secretion from CD4^+^ T cells and CD8^+^ T cells, we analyzed 2 sets of gene expression data from ovarian cancer tissues. Using the TCGA RNA-Seq data and our KOV microarray data, we found a significant correlation between CXCL13 and TGF-β1 gene expression (*P* < 0.05) (Figure 5A). The analysis of naive CD4^+^ T cells and CD8^+^ T cells isolated from the peripheral blood cells of a healthy donor and cultured with TGF-β showed that CD4^+^ T cells secreted more CXCL13 compared with CD8^+^ T cells. A TGF-β signal inhibitor (SB431542) suppressed the secretion of CXCL13 in a concentration-dependent manner (Figure 5B).

We previously reported that the TGF-β signaling pathway was activated in ovarian cancer (18), and we detected high concentrations of TGF-β1 in the conditioned medium of 3 different human ovarian cancer cell lines (Figure 5C). Using these conditioned media, we examined their effects on CXCL13 production in naive CD4^+^ and CD8^+^ T cells. In CD4^+^ T cells, CXCL13 secretion was promoted depending on the concentration of TGF-β1 in the conditioned medium, and it was suppressed in a concentration-dependent manner when incubated with the TGF-β signal inhibitor (SB431542) (Figure 5D).

We previously demonstrated that TGF-β and the blockade of IL-2 acted in synergy to induce CXCL13 (16, 17), and that IL-12 produced by DCs acted with TGF-β to induce various follicular helper T (Tfh) cell molecules in human CD4^+^ T cells (19). Therefore, we conducted similar experiments by adding inflammatory cytokines responsible for T cell differentiation to TGF-β.

We found that CXCL13 secretion was enhanced in CD4^+^ T cells under IL-2–restricted conditions and in CD8^+^ T cells under IL-12–enriched conditions (Figure 5E). The CXCL13 concentration in the culture supernatant showed a similar trend (Figure 5F). CXCL13-producing CD4^+^ cells had a PD-1^+^CXCR5^–^ phenotype (Figure 5G). These results suggest that the phase and TME in which CD4^+^ and CD8^+^ T cells produce CXCL13 are different; thus, CD4^+^ T cells may produce CXCL13 in TLS and CD8^+^ T cells in TILs (Figure 3, A and B).

### Mouse recombinant CXCL13 induces TLS in tumors and prolongs survival.

**Figure 6:**
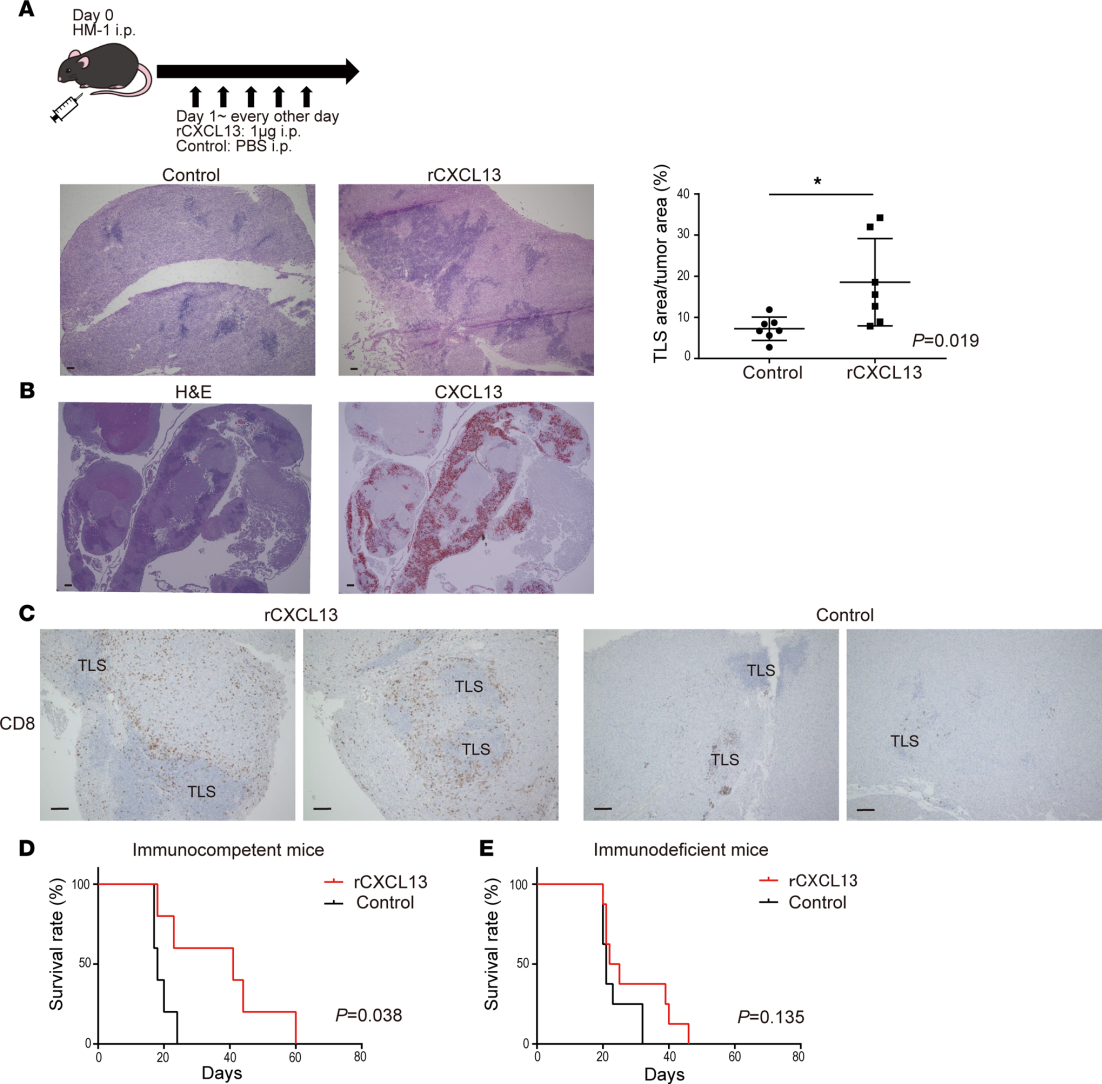
Mouse rCXCL13 induces TLS in tumors and prolongs survival. (**A**) Mouse rCXCL13 was administered i.p. to induce TLS in a mouse ovarian cancer model. Representative H&E images of TLS formed in an omental tumor. The area of TLS per tumor area was compared between the control group and the rCXCL13 treated group (*n* = 7, each). Statistical significance was determined by 2-tailed Student’s *t* test. *P < 0.05. (**B**) TLS induced by mouse rCXCL13 (H&E) and expression of mouse CXCL13 corresponding to TLS (RNA ISH, Fast RED). (**C**) CD8^+^ T cell IHC images (DAB) in the rCXCL13-treated group and control group. Scale bars: 100 μm. (**D** and **E**) The effect of rCXCL13 administration on the survival of tumor-bearing mice was compared between immunocompetent mice (**D**) and immunodeficient mice (**E**). Analyses were performed using Kaplan-Meier estimates and log-rank tests.

The effect of CXCL13 on TLS formation in tumors was analyzed using a mouse ovarian cancer model. A mouse ovarian cancer cell line OV2944-HM-1 (HM-1) was i.p. inoculated to B6C3F1 immunocompetent mice, and mouse recombinant CXCL13 (rCXCL13) was i.p. administered 5 times every other day starting from day 1, and TLS formed in the omental tumor were evaluated on days 10–12. The area of TLS per tumor area was significantly increased in the rCXCL13-treated group compared with the control group (Figure 6A). IHC revealed that mouse TLS, similar to human TLS, consisted mainly of CD19^+^ B cells, and that CD8^+^ T cells and CD4^+^ T cells were present in and around TLS. Furthermore, TLS contained many Ki-67^+^ immune cells, indicating that these structures were immunologically activated (Supplemental Figure 5A). Furthermore, CXCL13 was highly expressed and corresponding with TLS (Figure 6B). The administration of rCXCL13 markedly increased the infiltration of CD8^+^ T cells around the TLS (Figure 6C).

Next, we observed the effect of rCXCL13 administration on the survival of mice. In immunocompetent mice (B6C3F1), the survival time was significantly prolonged in the rCXCL13-treated group compared with the control group (Figure 6D). Because there was a correlation between CXCL13 and PD-1/PD-L1 gene expression (Supplemental Figure 5B), we hypothesized that CXCL13 had an adjuvant effect in HM-1 ovarian cancer models that were originally refractory to anti–PD-1/PD-L1 antibody therapy. However, no significant prognostic improvement was observed (Supplemental Figure 5C).

Furthermore, the administration of rCXCL13 to immunocompromised mice (nude mice) did not improve survival (Figure 6E). Consistent with the apparent increase in CD8^+^ T cells around the TLS in immunocompetent mice, CXCL13 contributed to their improved survival via immunity. These results indicate that CXCL13 induced TLS and TILs, indicating that CXCL13 and TLS might be new therapeutic targets for ovarian cancer.

## Discussion

Here, we showed that the presence of TLS was significantly associated with the infiltration of more than 2 lineages of lymphocytes and that, in any combination, the 2 lineages of lymphocytes might cooperate to improve the prognosis of ovarian cancer. It was reported that tumor-infiltrating B cells contributed to tumor growth and progression through the production of cytokines, such as IL-10, that inhibit antitumor immunity, although the functional role of B cells in cancer is poorly understood (20). Recently, there have been increasing numbers of reports that the presence of B cells, especially those associated with TLS, may improve cancer outcomes (10, 12–14). Tumors containing CD8^+^ T cells and B cell lineages were associated with improved prognosis in melanoma, sarcoma, and ovarian cancer (12, 13, 21). In this study, we found a strong correlation between the infiltrated numbers of CD8^+^ T cells and B cell lineages, and we confirmed the presence of TLS in 94% of patients with tumors containing high numbers of infiltrated T and B cells (Supplemental Figure 1D).

Whether tumor-associated TLS are formed in response to a series of chronic inflammation or whether they are induced as a tumor antigen–specific immune response has not been fully established. De Chaisemartin et al. demonstrated that TLS provided the specialized vasculature and chemoattractants necessary for T cell infiltration into non–small cell lung cancer (NSCLC) (22). The TCR repertoire analysis of NSCLC showed that the expansion of T cell clones in the tumor bed and peripheral blood correlated with the density of tumor-associated TLS (23). In this study, TCR repertoire analysis of the TLS and tumor regions revealed that oligoclonal expansion occurred in TLS and that the same clone was highly amplified in the tumor. These data suggest that the recognition of antigens occurs in TLS and that the effector T cell clones amplified by the TLS infiltrate into tumors, both of which might provide evidence that TLS promotes immune responses in TME. The presence of TLS provides a site for the infiltration and proliferation of T and B cell lineages, and the interaction between cellular and humoral immunity may lead to improved prognosis.

High CXCL13 gene expression was associated with disease activity and pathogenicity in autoimmune diseases, such as RA (24–26), and with patients’ prognosis in several types of cancer (7, 27, 28), implying the strong involvement of CXCL13-dependent TLS formation. In this study, we found that CXCL13 gene expression was a prognostic factor for ovarian cancer and was associated with the presence of TLS. Only CXCL13 was correlated with the infiltration of all TIL subsets, TLS presence, and prognosis; however, it did not correlate with the cytokines CCL19 and CCL21, which are also involved in TLS formation.

We previously reported that CXCL13-producing PD-1^hi^CXCR5^–^CD4^+^ T cells have an important role in the function of TLS in RA (16, 17). Although FDCs are the main source of CXCL13 in SLOs (29), the origin of CXCL13 in tumor associated TLS depends on the type of cancer. CXCL13 was secreted by PD-1^hi^CD8^+^ T cells in lung cancer (30), by CD103^+^CD8^+^ cells in ovarian cancer (31), and by CXCR5^–^PD-1^hi^CD4^+^ follicular helper like T cells in breast cancer (32) and nasopharyngeal cancer (33). In this study, CXCL13 was predominantly expressed by CD4^+^ T cells in TLS, whereas it was expressed by CD8^+^ and CD4^+^ T cells in TILs. In some TLS, the expression of CXCL13 by CD4^+^ T cells was replaced by CD21^+^ FDCs. Sequential stages of the development in tumor-associated TLS were observed in lung cancer. Siliņa et al. defined 3 types of TLS: early TLS without FDCs or germinal centers (GC), primary follicle-like TLS (PFL-TLS) with an FDC network and lacking GC, and secondary follicle-like TLS (SFL-TLS) with an FDC network and GC formation (34). Our data suggest that CXCL13-producing CD4^+^ T cells are important primary producers of CXCL13 in the early stages of TLS when FDCs are not present. FDCs emerge from ubiquitous perivascular mesenchymal cells expressing platelet-derived growth factor receptor β (35). CXCL13 itself directly induces lymphotoxin (LT) production by naive B cells, and this CXCL13/LT pathway is crucial for FDC differentiation (36–38). We consider that FDC becomes the main source of CXCL13 in TLS after the FDC network is formed, similar to that in SLOs.

The CXCL13-producing PD-1^hi^CXCR5^–^CD4^+^ T cells that we reported in RA (16, 17) do not express CXCR5, a marker typical of Tfh cells. In the current study, the CD4^+^ T cells in which CXCL13 expression was induced were PD-1^hi^CXCR5^–^. The CXCL13-producing CD4^+^ T cells reported in breast cancer (32) and nasopharyngeal cancer (33) had similar characteristics. The comprehensive analysis of blood and synovial samples of RA patients was used to propose a pathogenic PD-1^hi^CXCR5^–^CD4^+^ subset as peripheral helper T (Tph) cells (39, 40). Tph cells express factors that enable B cell help, including IL-21, CXCL13, and ICOS. Similar to PD-1^hi^CXCR5^+^ Tfh cells, Tph cells induce plasma cell differentiation in vitro through IL-21 secretion and SLAMF5 interactions (39). Li et al. also reported that CXC13-producing CD4^+^ T cells with a Tph phenotype secreted IL-21 and activated B cells through interactions with CD84 in nasopharyngeal cancer (33). In this context, CXCL13-producing CD4^+^ T cells not only promote the initial formation of TLS, but may also support antitumor antibody responses by B cells. Evidence for this was shown in our study, where B cell lineages were clearly increased in patients with TLS and were associated with an improved prognosis. However, further research related to the colocalization of antigen-specific B cells with Tph cells in TLS is warranted at the molecular level.

We previously reported the TGF-β concentration–dependent promotion of the differentiation of CXCL13-producing CD4^+^ T cells under inflammatory conditions involving TGF-β (16, 17). Resident fibroblasts and macrophages, as well as infiltrating Tregs, produce TGF-β locally (41). Previously, we reported that the TGF-β signaling pathway was activated in advanced ovarian cancer and promoted tumor progression and metastasis (18). Indeed, high levels of TGF-β1 were detected in the conditioned medium of DK-09, a cell line that we established from the ascites of a recurrent multidrug-resistant HGSC patient. In this study, we performed a CXCL13 induction assay using PBMCs from a healthy donor and found that TGF-β promoted CXCL13 secretion, although there was a significant difference in response to TGF-β between CD4^+^ T cells and CD8^+^ T cells. Furthermore, CXCL13 production was enhanced in CD4^+^ T cells under an IL-2–restricted environment and in CD8^+^ T cells under an IL-12–rich environment. In addition to TGF-β, IL-2 is involved in the differentiation of CD4^+^ T cells. Gu-Trantien et al. reported that IL-2 deprivation was critical for the production of CXCL13 and the accumulation of activated Tregs in parallel with CXCL13^+^ CD4^+^ TIL in breast cancer (32). IL-12, produced by mature DCs and macrophages, has a strong effect on T cell activation and is considered important as a bridge between the innate and acquired immune systems (42–44). We showed that TGF-β plus IL-12 did not enhance CXCL13 production by CD4^+^ T cells, whereas it significantly promoted CXCL13 secretion by CD8^+^ T cells. Duraiswamy et al. reported the presence of a niche where CD8^+^ TILs clustered with DCs and macrophages in the tumor locus of ovarian cancer, and that PD-1^+^CD8^+^ TILs costimulated with CD28 strongly expressed CXCL13 (45). Their study may provide an explanation whereby activated DCs and macrophages in these niches provide a supply of IL-12. Of note, our previous reports confirmed that adding TNF, IL-1β, and IL-6, which are known to be elevated in the TME of ovarian cancer, to TGF-β had no effect on CXCL13 secretion (17).

CD8^+^ T cells producing CXCL13 were reported in lung cancer (30) and ovarian cancer (31, 45), whereas an analysis in nasopharyngeal cancer reported that most subsets of T cells producing CXCL13 were CD4^+^ T cells (33). We showed that, in ovarian carcinoma, CD8^+^ T cells were dominant among TILs, whereas CD4^+^ cells were dominant in the TLS. We further showed that, in vitro, culture conditions to promote CXCL13 secretion were different between CD4^+^ and CD8^+^ T cells. Thus, our study shows that CXCL13 expression by CD4^+^ and CD8^+^ T cells is regulated differently; therefore, the source of CXCL13 expression differs between TLS (CD4^+^ T cell dominant) and TILs (CD8^+^ T cell dominant).

Finally, we evaluated whether TLS was induced by CXCL13. In a mouse model of spontaneously developing gastric cancer by activated STAT3 signaling, chemokines CXCL13, CCL19, and CCL21 were induced simultaneously with tumorigenesis and TLS formation (46). We administered mouse rCXCL13 one day after tumor inoculation and succeeded in inducing TLS. CXCL13 was highly expressed and corresponding with TLS. CXCL13 signals B cells to enhance LT production (36, 47), and the exogenous CXCL13 may have promoted a positive feed-forward loop. In the CXCL13-treated group, the infiltration of CD8^+^ T cells around the TLS was clearly increased, and the survival time of tumor-bearing mice was also prolonged. Direct antitumor effects by CXCL13 were also observed in a colon cancer model. However, tumor growth was accelerated in CXCR5 or Rag1-KO mice (28). The CXCL13 axis is a functional part of the relevant immune control, and the TME can be altered by inducing CXCL13 and TLS. Accordingly, our results clearly demonstrate that the induction of CXCL13 and TLS has potential as an immune-modulatory target for ovarian cancer. This study had some limitations. First, we did not directly prove that CXCL13 secreted from CD4^+^ T cells induced TLS in the mouse ovarian cancer model. It is well established that, unlike human CD4^+^ T cells, murine CD4^+^ T cells do not secrete CXCL13 (17, 48). Therefore, we could not assess the significance of CXCL13 derived from CD4^+^ T cells on the formation of TLS in our mouse cancer models. Alternatively, we demonstrated that the injection of CXCL13 induced TLS formation accompanied by the inhibition of tumor growth in mouse models, and that CD4^+^ T cells were the major source of CXCL13 in human ovarian cancer TLS.

Taken together, CXCL13 is a strong prognostic factor for ovarian cancer and is highly involved in the formation of TLS. CXCL13-producing CD4^+^ T cells induced by tumor environments such as TGF-β are important for the initial formation of TLS. The presence of TLS recruits various lymphocytes and enhances the infiltration of B cell lineages and CD8^+^ T cells in ovarian cancer, leading to antitumor immunity. The strong interaction between humoral and cellular immunity in the antitumor response was revealed, and the possibility of TLS-mediated interactions was demonstrated. In vivo experiments revealed that the TME can be altered by inducing CXCL13 and TLS, which might be an important immunomodulatory method to enhance antitumor immunity.

## Methods

### Human samples.

In total, 62 patients with HGSC who underwent primary surgery at Kyoto University Hospital between 1997 and 2015 after approval of the study protocol by the Institutional Ethical Committee were enrolled. We obtained another 34-patient cohort that underwent primary surgery of HGSC at Kindai University Hospital between 2009 and 2016 after approval of the study protocol by the Institutional Ethical Committee. Their clinical characteristics are described in Supplemental Table 1. Patients who received chemotherapy or radiation therapy prior to surgery were excluded. Four other patients with TLS who underwent initial surgery at the National Hospital Organization Kyoto Medical Center between 2017 and 2019 were also included in the study.

### IHC analysis.

FFPE specimens were obtained from the above patients. IHC staining was performed using the streptavidin-biotin-peroxidase method as previously described (4, 6). The samples were incubated with mouse anti-CD8 (clone C8/144B, catalog 413201, Nichirei Biosciences), rabbit anti-CD4 monoclonal antibody (clone SP35, catalog 104R-14, 1:100 dilution, Cell Marque), mouse anti-CD20 monoclonal antibody (clone L26, catalog NBP2-44743, 1:500 dilution, Novus Biologicals), rabbit anti-CD38 monoclonal antibody (clone SP149, catalog ARG52764, 1:100 dilution, Arigo Biolaboratories), and mouse anti-CD21 antibody (clone 1F8, catalog NBP1-22527, 1:10 dilution, Novus Biologicals).

### Evaluation of IHC.

Two independent investigators trained in the pathology of ovarian cancer and blinded to the clinical data examined the H&E staining and IHC slides. The presence of TLS was determined based on (a) typical cell aggregation assessed by H&E staining and (b) accumulation of CD20^+^ B cells inside the aggregation. When we analyzed TILs without TLS, we selected at least 5 HPFs distant from the TLS areas, and we counted the numbers of CD8^+^, CD4^+^, and CD20^+^ cells in the field. Because the distribution of these lymphocytes was divided into 2 groups around the median by kernel density estimation, counts above the median were defined as CD8^hi^, CD4^hi^, and CD20^hi^ tumors. Tumor-infiltrated CD38^+^ plasma cells were graded according to their intensity and fraction of positive cells as 0, 1, 2, or 3 (plasma cell score) according to previous reports (21, 49). Cases with scores of 0 and 1 were defined as plasma cell–low tumor, and cases with scores of 2 and 3 were defined as plasma cell–high tumors.

### Detection of CXCL13 mRNA by RNA ISH.

We assessed CXCL13 by RNA ISH (RNAscope 2.5 HD Reagent kit [RED]; Advanced Cell Diagnostics). FFPE tissue sections were deparaffinized in xylene and subsequently dehydrated in an ethanol series. Tissue sections were incubated in target retrieval reagent at 100°C for 15 minutes and were then treated with protease at 40°C for 30 minutes. Hybridization with Hs-CXCL13 (for human) or Mm-Cxcl13 (for mouse) probes at 40°C for 2 hours, and the amplifier and visualization (Fast RED) procedures, were performed in accordance with the manufacturer’s instructions. For multiplex detection using FFPE, an RNAscope Fluorescent Multiplex Reagent kit v2 (Advanced Cell Diagnostics) was used. Double staining was performed for CXCL13 and CD8, CXCL13 and CD4, and CXCL13 and CD21 (CR2). The target probes were Hs-CXCL13 (C1), Hs-CD4 (C2), Hs-CD8A (C2), and Hs-CR2 targeting 269-1303 of NM_001006658.3 (C2). CXCL13 was detected with Opal 690 (1:1000 dilution, PerkinElmer), and CD4, CD8, and CR2 were detected with Opal 570 (1:1500 dilution, PerkinElmer). Fluorescence images were captured using a fluorescence microscope BZ-X800E (KEYENCE), and the colocalization of CXCL13 with various immune cells was quantified using BZ-H4C/hybrid cell count software (KEYENCE).

### HGSC gene expression analysis.

HGSC specimens obtained from 28 patients who underwent primary surgery at Kyoto University Hospital from 1997 to 2012 were prepared for gene expression microarray analysis (KOV). The data were previously deposited in the Gene Expression Omnibus (accession nos. GSE39204 and GSE55512; https://www.ncbi.nlm.nih.gov/gds/).

The gene expression profile of the TCGA-OV RNA-Seq data set (*n* = 217) from the TCGA Data Portal (http://cancergenome.nih.gov, illuminahiseq_rnaseqv2_Level_3_RSEM_genes_normalized_data files obtained and merged on Oct. 19, 2015) was used for survival analysis and correlation testing among CXCL13, TGF-β1, PDCD1, and CD274.

### Gene expression and infiltrating immune cells analysis.

Raw gene expression microarray data using Affymetrix HT_HG-U133A from TCGA ovarian serous cystadenocarcinoma samples were obtained from the GDC legacy archive (https://portal.gdc.cancer.gov/legacy-archive/) in the form of CEL files (*n* = 522). Gene expression values were calculated by normalization using the RMA method with R package “affy” (http://www.R-project.org). Subsequently, the relative abundance of 22 immune cell types for each sample was estimated using CIBERSORT (http://cibersort.stanford.edu/).

### TCR and BCR repertoire analysis.

TLS and tumor areas were identified by microscopy and macrodissected independently from FFPE sections (Supplemental Figure 1C). RNA was extracted using NucleoSpin total RNA FFPE (Takara Bio) according to the manufacturer’s instructions. Sequencing of the TCRα (TRA) and BCR IgG heavy chain loci were performed at Repertoire Genesis Incorporation using an unbiased amplification method with MiSeq (Illumina). Data processing, assignment, and aggregation were performed using a repertoire analysis software program, Repertoire Genesis (RG), provided by Repertoire Genesis Incorporation. RG assigns TRV and TRJ alleles to queries and then generates CDR3 sequences, finally aggregating their combination patterns.

### Induction assay of CXCL13 in CD4^+^ and CD8^+^ T cells.

PBMCs from healthy donors were collected using Lymphocyte Separation Solution 1.077 (Nacalai Tesque). Blood CD8^+^ T cells were isolated with CD8 MicroBeads (human; Miltenyi Biotec). Blood CD4^+^ T cells were purified with naive CD4^+^ T cell isolation kit II (human; Miltenyi Biotec) through a magnetic column.

Human T cells were differentiated for 6–7 days in a humidified 5% CO_2_ incubator at 37°C with IMDM (Thermo Fisher Scientific) supplemented with 10% FBS (Thermo Fisher Scientific), 100 units/mL penicillin and streptomycin (Thermo Fisher) under stimulation with 5 μg/mL plate-bound CD3 monoclonal antibody (clone OKT3, Thermo Fisher Scientific) and 10 μg/mL CD28 monoclonal antibody (clone CD28.2, Thermo Fisher Scientific) in the presence of 10 ng/mL TGF-β1 (Cell Signaling Technology) unless otherwise described. Human T cells were also cultured under conditions where 5 μg/mL neutralizing anti–IL-2 antibody (catalog MAB202, R&D Systems) or 10 ng/mL IL-12 (PeproTech,) was added.

Human T cells were cultured under CD3/CD28 stimulation using conditioned medium from the human serous ovarian cancer cell lines: OVCA420, OVCA433, and DK-09. OVCA420 and OVCA433 were provided by Susan K. Murphy, of Duke University (Durham, North Carolina, USA). DK-09 is a cell line that we established from the ascites of a patient with recurrent ovarian cancer (see Supplemental Methods). To block the TGF-β signals, a TGF-β signal inhibitor, SB431542 (Stemgen) was added at 0.5 and 5 μM.

### Flow cytometry.

For intracellular staining, cells were cultured for 4 hours with 4 μM monensin (Sigma-Aldrich), fixed, and stained with eBioscience Intracellular Fixation & Permeabilization Buffer (Thermo Fisher Scientific) and antibodies for intracellular molecules. Fixable Viability Dye eFluor 506 (Thermo Fisher Scientific) was used to exclude dead cells. APC-conjugated anti-CXCL13 antibody (clone 53610, catalog IC801A, R&D Systems), BV421 conjugated rat anti-human CXCR5 (clone RF8B3, catalog 562747, BD Biosciences), and PE-conjugated anti-human CD279 (PD-1) (clone EH12.2H7, catalog 329906, BioLegend) were used. Data were acquired using a MACS Quant (Miltenyi Biotec) and were analyzed with FlowJo 10.0 (Tree Star Inc.).

### ELISA.

The concentrations of CXCL13 and TGF-β1 in the supernatant were measured with a Human CXCL13/BLC/BCA-1 Quantikine ELISA Kit (R&D Systems) and TGF-β1 Human ELISA Kit (Thermo Fisher Scientific) according to the manufacturer’s instructions.

### Cell lines and tumor models.

The HM-1 mouse ovarian cancer cell line was purchased from RIKEN BioResource Center and cultured as described (50). Throughout the study, we used HM-1 cell lines passaged fewer than 20 times and regularly tested for mycoplasma contamination. Authentication of these cells with short tandem repeat analysis was not performed because they were derived from mice. Female B6C3F1 (C57BL6 × C3/He F1) mice and nude mice (BALB/C-nu: CAnN.Cg-*Foxn1^nu^*/CrlCrlj) were purchased from Charles River Laboratories and were maintained under specific pathogen–free conditions.

In total, 1 × 10^6^ HM-1 cells were inoculated i.p. into B6C3F1 mice. Mouse rCXCL13 (R&D Systems) treatment was initiated 1 day after the tumor inoculation and administered i.p. at 1 μg/mouse every other day 5 times. Control mice received PBS i.p. Then, 10–12 days after inoculating the tumor, mice were euthanized with carbon dioxide, and the formation of TLS in omental tumors was analyzed.

A total of 2.5 × 10^5^ HM-1 cells was inoculated i.p. into B6C3F1 and nude mice. Similarly, rCXCL13 (1 μg/mouse) and anti–PD-L1 antibody (200 μg/mouse; clone 10F.9G2, catalog BE0101, Bio X Cell) were i.p. administered 5 times every other day starting from day 1 and day 3, respectively, after tumor implantation. Anti–PD-L1 antibody (clone 10F.9G2) and rat IgG antibody (clone LFT-2, catalog BE0090, Bio X Cell) as negative controls were used.

### IHC analysis of mouse tumors.

Mouse tumor cryosections (6 μm thick) were stained with anti-CD4 (clone H129.18, catalog 550278, 1:200 dilution, BD Pharmingen/BD Biosciences), anti-CD8 (clone YTS169.4, catalog ab22378, 1:200 dilution, Abcam), anti-CD19 (clone 6D5, catalog ab25232, 1:200 dilution, Abcam), and anti–Ki-67 (clone SolA15, catalog 14-5698-80, 1:100 dilution, Thermo Fisher Scientific) antibodies as previously described (51). Mouse FFPE specimens were stained with anti-CD8 antibody (clone EPR20305, catalog ab209775, 1:2000 dilution, Abcam).

### Statistics.

Results are shown as the mean ± SEM from at least 3 independent experiments unless otherwise stated. A *P* value of less than 0.05 was considered statistically significant. Significance was calculated using the 2-tailed Student’s *t* test, and correlation between groups was determined by Spearman’s correlation test. The log-rank test was used for overall survival analysis unless otherwise described. Statistical analyses were performed using GraphPad Prism 7 and R (version 4.0.5).

### Study approval.

This study was approved by Kyoto University Graduate School and Faculty of Medicine, Institutional Ethical Committee (Kyoto, Japan), Ethics Committee of Kindai University Faculty of Medicine (Osaka, Japan), and Institutional Ethical Committee of the National Hospital Organization Kyoto Medical Center (Kyoto, Japan). Informed consent was obtained in the form of opt-out on the website for the patients at Kyoto University and Kindai University (https://kyoto.bvits.com/rinri/publish_document.aspx?ID=1747 and https://www.med.kindai.ac.jp/laboratory/obstetrics_and_gynecology/research/opt_out/). Written informed consent was obtained from the patients at Kyoto Medical Center. Animal experiments were approved by the Kyoto University Animal Research Committee. This study was conducted according to Declaration of Helsinki principles.

## Author contributions

MU designed and performed the experiments, analyzed the data, and wrote the manuscript. JH and HY designed and directed the study and edited the manuscript. ST performed statistical analyses and commented on the manuscript. HN and KA provided the clinical data. YH assisted with the experiments. AU, HS, YF, MT, K. Yamanoi, KA, K. Yamaguchi, TB, NM, AY, HU, and MM provided advice on the experiments and commented on the manuscript.

## Supplementary Material

Supplemental data

## Version 1 05/12/2022

In-Press Preview

## Version 2 06/22/2022

Electronic publication

## Version 3 06/28/2022

pdf now loads
