# Supplementary material for: CXCL13-producing CD4+ T cells accumulate in the early phase of tertiary lymphoid structures in ovarian cancer
Source: JCI Insight. 2022 Jun 22;7(12):e157215. doi: 10.1172/jci.insight.157215 (PMC9309049; doi:10.1172/jci.insight.157215)
Supplement: Supplemental data [file jciinsight-7-157215-s265.pdf]

Supplemental methods

Establishment of a patient derived cell line, DK-09

The patient was diagnosed with peritoneal carcinoma stage IIIB, and the histology indicated high grade serous carcinoma. The patient underwent primary debulking surgery and six cycles of dose-dense paclitaxel/carboplatin as adjuvant chemotherapy. The patient was treated with gemcitabine/carboplatin + bevacizumab and pegylated liposomal doxorubicin after recurrence, but they were ineffective. When cell-free and concentrated ascites reinfusion therapy (CART) was performed for the palliation of symptoms, a sample of ascites was obtained with the patient's informed consent. The cells were collected by centrifugation, lysed with ACK lysing buffer (LONZA, Basel, Switzerland) to remove red blood cells, and treated with Liberase (37°C for 30 minutes) (Roche, Basel, Switzerland). CD45-MACS beads (Miltenyi Biotec) were added and $CD45^+$ cells were removed by the column method.

Flow cytometry was used to confirm the cells were negative for CD45 after the removal of $CD45^+$ cells. Anti-fibroblast antibody was used to confirm the absence of fibroblasts. We confirmed that approximately 60% of the cells were positive for FOLR1, which is positive in most ovarian cancers. The cells were then passaged in RPMI + 20% fetal bovine serum, and autonomous growth was observed. In Passage 28 cells, 98.7% of the cells were CD45-negative and 77% of the cells were FOLR1-positive, and these cells were used in subsequent experiments.

Supplemental Table 1. Patients' characteristics

	Kyoto Univ. cohort	Kindai Univ. cohort	Total
Histology	High grade serous carcinoma		
No. of cases	62	35	97
Age (median, years)	56.5	63	60
(range)	(35-82)	(42-84)	(35-84)
Stage I	7	7	14
Stage II	5	3	8
Stage III	44	17	61
Stage IV	6	8	14
TLS positive cases	39	22	61
(%)	62.9	62.8	62.9
Median follow up (month)	76	52	59
(range)	(8-196)	(1-120)	(1-196)

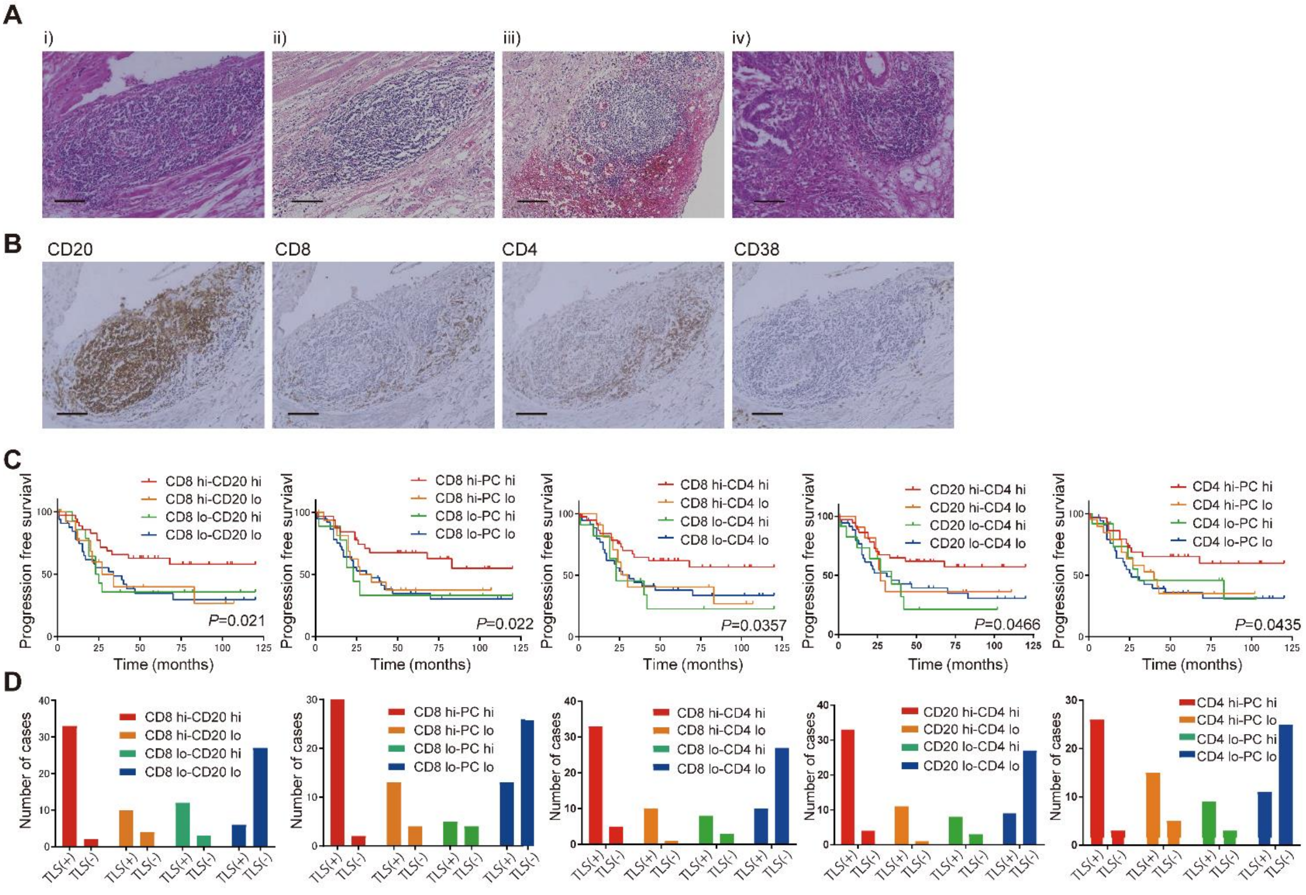

Supplemental Figure 1. TLS and various lymphocyte infiltrations

(A) Representative H&E images of TLS. **(B)** Distribution of various lymphocytes in TLS. IHC images (DAB) of CD20, CD8, CD4, and CD38 in A(i). Scale bars indicate 100 μm in (A) and (B). **(C)** Progression free survival of patients based on the combination of two lineages of infiltrating lymphocytes (CD8 hi-CD20 hi: n=35, CD8 hi-CD20 lo: n=14, CD8 lo-CD20 hi: n=15, CD8 lo-CD20 lo: n=33), (CD8 hi-PC hi: n=32, CD8 hi-PC lo: n=17, CD8 lo-PC hi: n=9, CD8 lo-PC lo: n=39), (CD8 hi-CD4 hi: n=38, CD8 hi-CD4 lo: n=11, CD8 lo-CD4 hi: n=11, CD8 lo-CD20 lo: n=37), (CD20 hi-CD4 hi: n=37, CD20 hi-CD4 lo: n=12, CD20 lo-CD4 hi: n=11, CD20 lo-CD4 lo: n=36), and (CD4 hi-PC hi: n=29, CD4 hi-PC lo: n=20, CD4 lo-PC hi: n=12, CD4 lo-PC lo: n=36). **(D)** Distribution of TLS in relation to the infiltration pattern of immune cells in tumors. Tumors were considered high (hi) for $CD8^+$ T cells, $CD20^+$ B cells and $CD4^+$ T cells if their score was above the median. Tumors were divided into two groups for PC by plasma cell score (0–1 n=56, 2–3 n=41), with 0–1 defined as PC low (lo) and 2–3 as PC hi in (C), and (D).

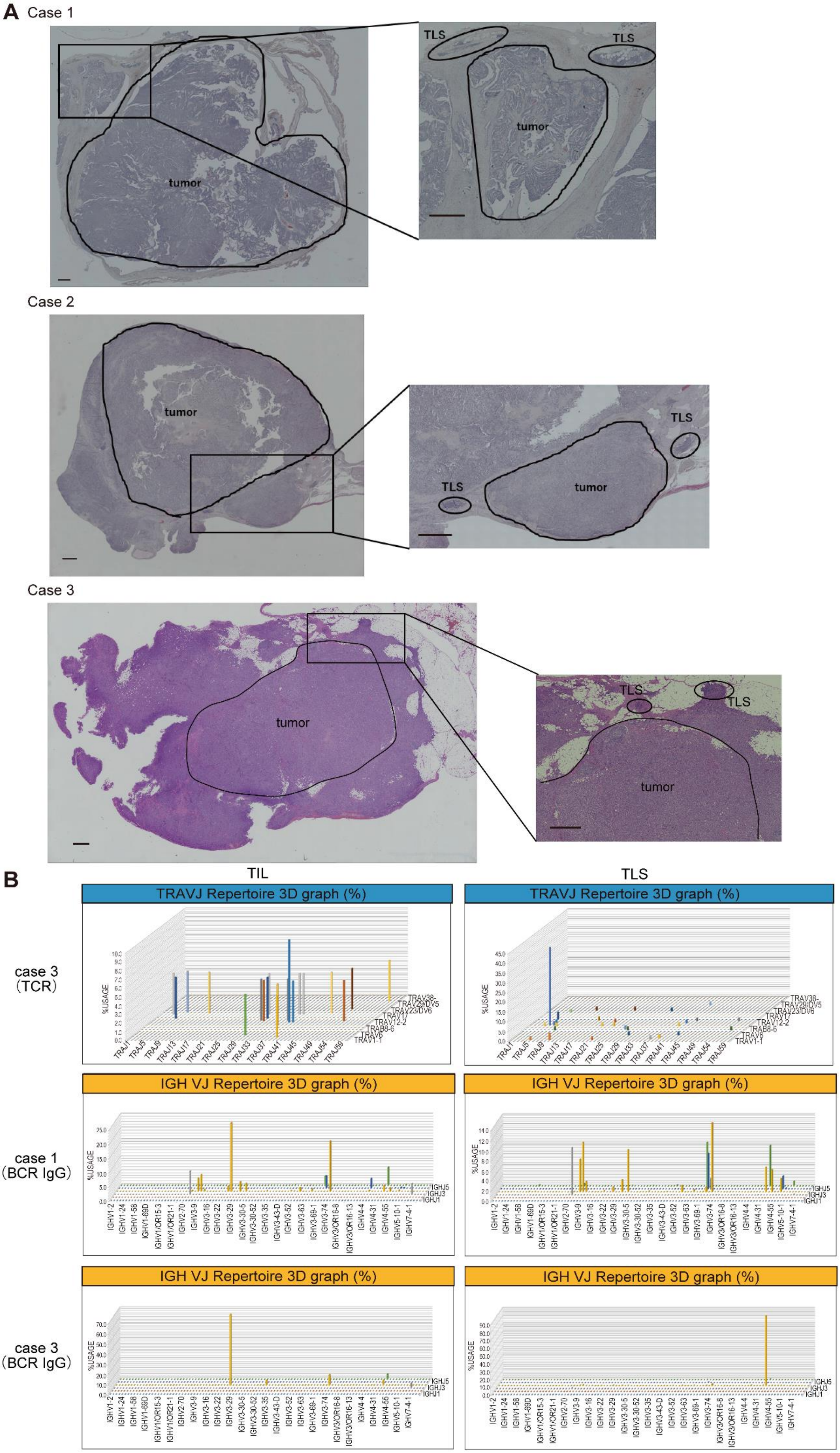
A
Case 1
TLS
TLS
tumor
tumor
Case 2
tumor
TLS
TLS
tumor
Case 3
tumor
TLS
TLS
tumor
B
TIL
TLS
TRAVJ Repertoire 3D graph (%)
TRAVJ Repertoire 3D graph (%)
case 3
(TCR)
%USAGE
IGH VJ Repertoire 3D graph (%)
IGH VJ Repertoire 3D graph (%)
case 1
(BCR IgG)
IGH VJ Repertoire 3D graph (%)
IGH VJ Repertoire 3D graph (%)
case 3
(BCR IgG)

Supplemental Figure 2. Repertoire analysis separating TLS and tumor site (TIL)

(A) As shown in the figure, the tumor area and TLS area were separated by macrodissection. The tumor region was analyzed as TIL and the TLS region as TLS for repertoire analysis. Scale bars indicate 1000 µm. **(B)** TCR and BCR repertoire analysis separating TLS and TIL regions. The horizontal axis represents the J gene, the depth represents the V gene, and the vertical axis represents the frequency of usage by TCR repertoire analysis. The horizontal axis represents the V gene, the depth represents the J gene, and the vertical axis represents the frequency of usage by BCR repertoire analysis. BCR analysis was difficult to perform in Case 2.

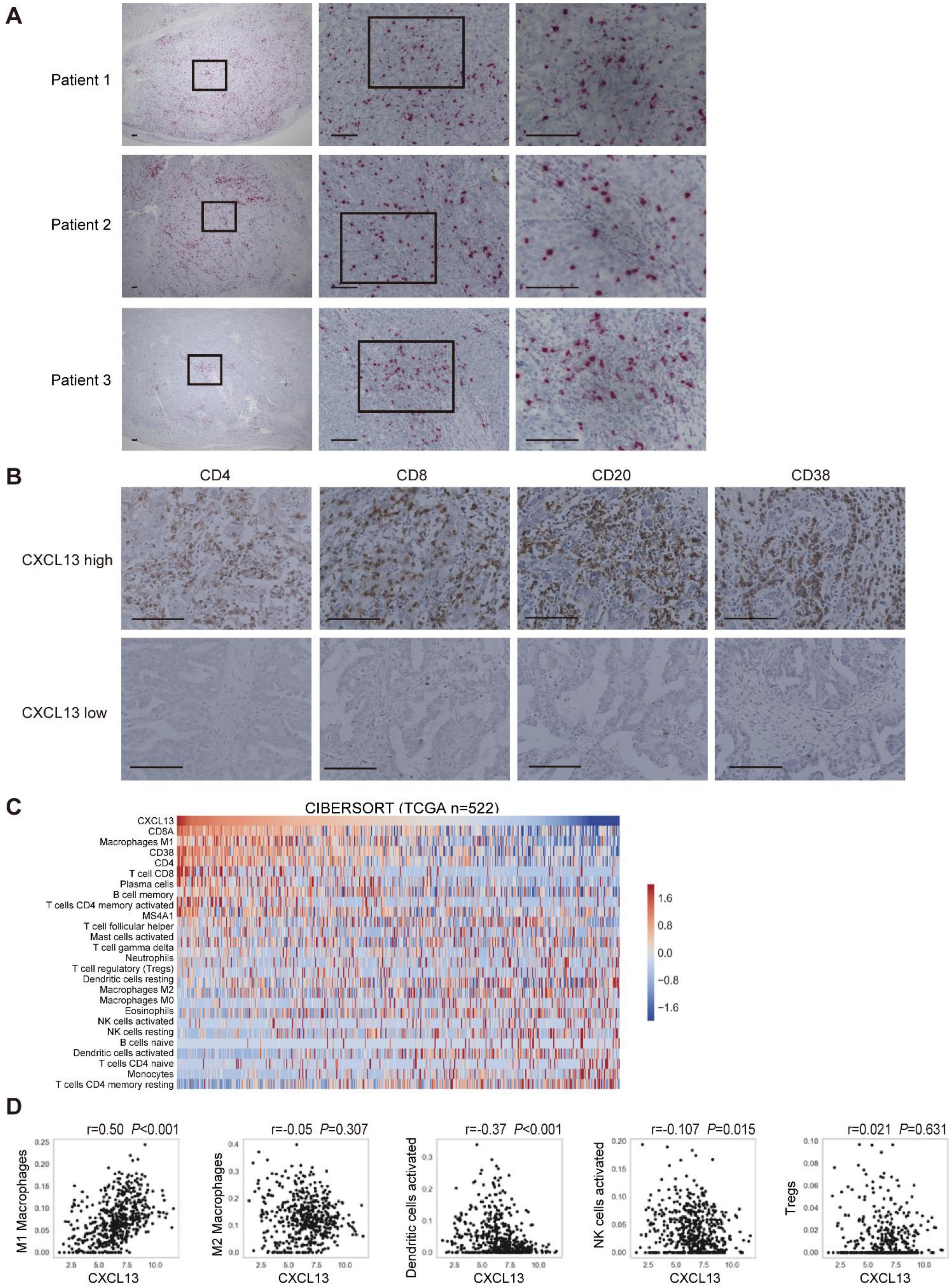

A
Patient 1
Patient 2
Patient 3
B
CD4
CD8
CD20
CD38
CXCL13 high
CXCL13 low
C
CIBERSORT (TCGA n=522)
CXCL13
CD8A
Macrophages M1
CD38
CD4
T cell CD8
Plasma cells
B cell memory
T cells CD4 memory activated
MS4A1
T cell follicular helper
Mast cells activated
T cell gamma delta
Neutrophils
T cell regulatory (Tregs)
Dendritic cells resting
Macrophages M2
Macrophages M0
Eosinophils
NK cells activated
NK cells resting
B cells naive
Dendritic cells activated
T cells CD4 naive
Monocytes
T cells CD4 memory resting
1.6
0.8
0.0
−0.8
−1.6
D
r=0.50 P<0.001
M1 Macrophages
r=-0.05 P=0.307
M2 Macrophages
r=-0.37 P<0.001
Dendritic cells activated
r=-0.107 P=0.015
NK cells activated
r=0.021 P=0.631
Tregs
CXCL13

Supplemental Figure 3. CXCL13 expression and intratumor infiltrating lymphocytes

(A) CXCL13 expression in tumor infiltrating immune cells by RNA ISH (Fast RED). Representative three cases with a high expression of CXCL13 in immune cells. Scale bars indicate 100 μm. **(B)** CXCL13 gene expression and distribution of several types of tumor-infiltrating immune cells. The upper row shows a case with high CXCL13 gene expression and the lower row shows a case with low CXCL13 gene expression. Scale bars indicate 100 μm. **(C)** The distribution of infiltrating immune cells into the tumor site and CXCL13 gene expression using CIBERTSORT (n=522). Overall view of the image shown in Figure 2D. **(D)** Correlation of CXCL13 gene expression with M1- or M2-macrophages, activated dendritic cells, activated natural killer cells, or regulatory T cells (Tregs) in CIBERSORT. Correlation was determined by Spearman's correlation test.

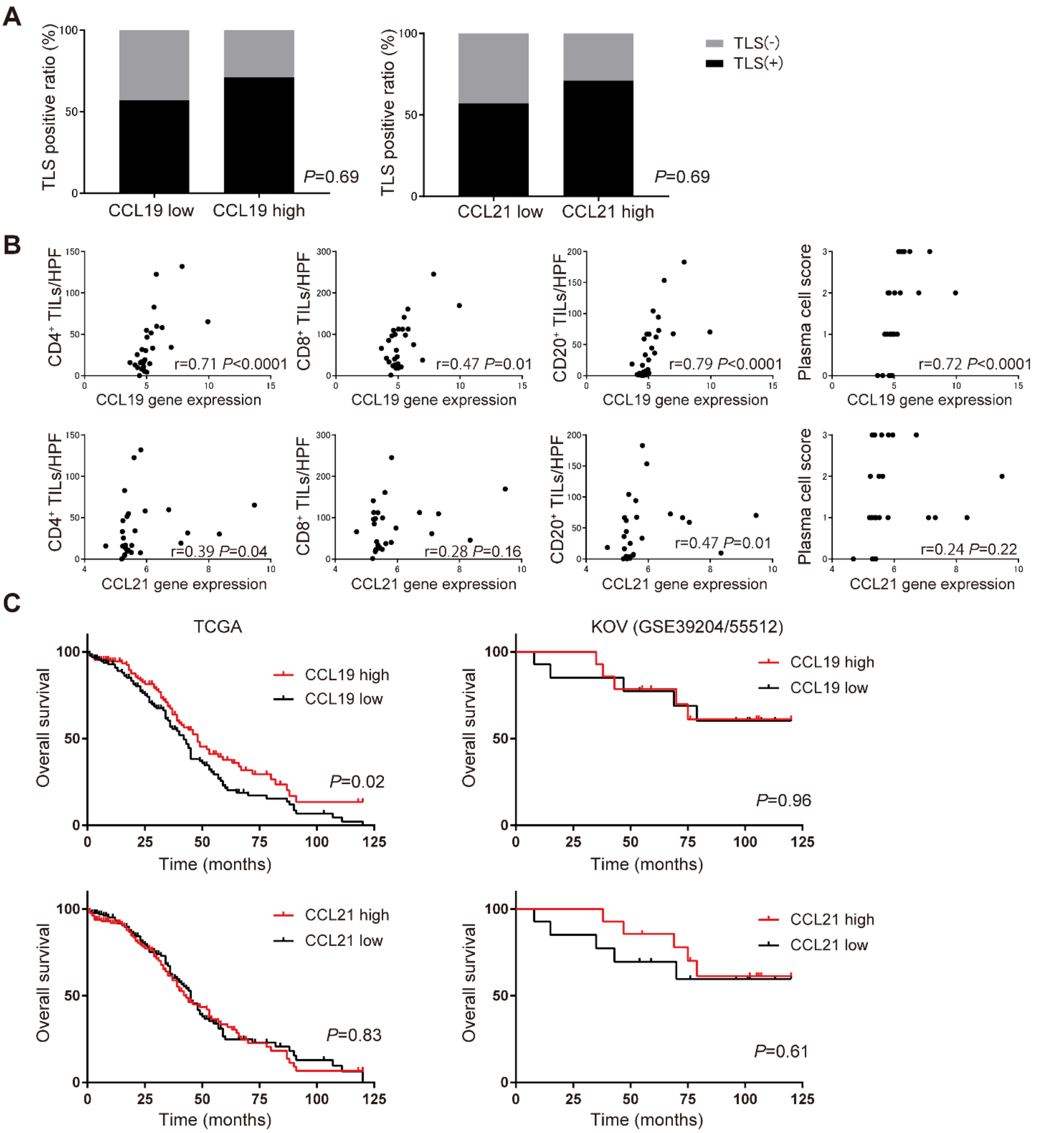

Supplemental Figure 4. Relationship between CCL19 or CCL21 and TLS formation, TIL, and prognostic impact

(A) TLS presence ratio based on CCL19 or CCL21 gene expression. Analysis by Fisher's exact test in 28 cases with microarray data. **(B)** Characterization of the immune infiltrate in tumors according to CCL19 or CCL21 gene expression (n=28). Correlations were determined by Spearman's correlation test. **(C)** Overall survival of patients with HGSC by CCL19 or CCL21 gene expression (TCGA n=217, KOV n=28). Patients were defined as CCL19 or CCL21 high if CCL19 or CCL21 gene expression was above the median value. Analyses were performed with Kaplan-Meier estimates and log-rank tests.

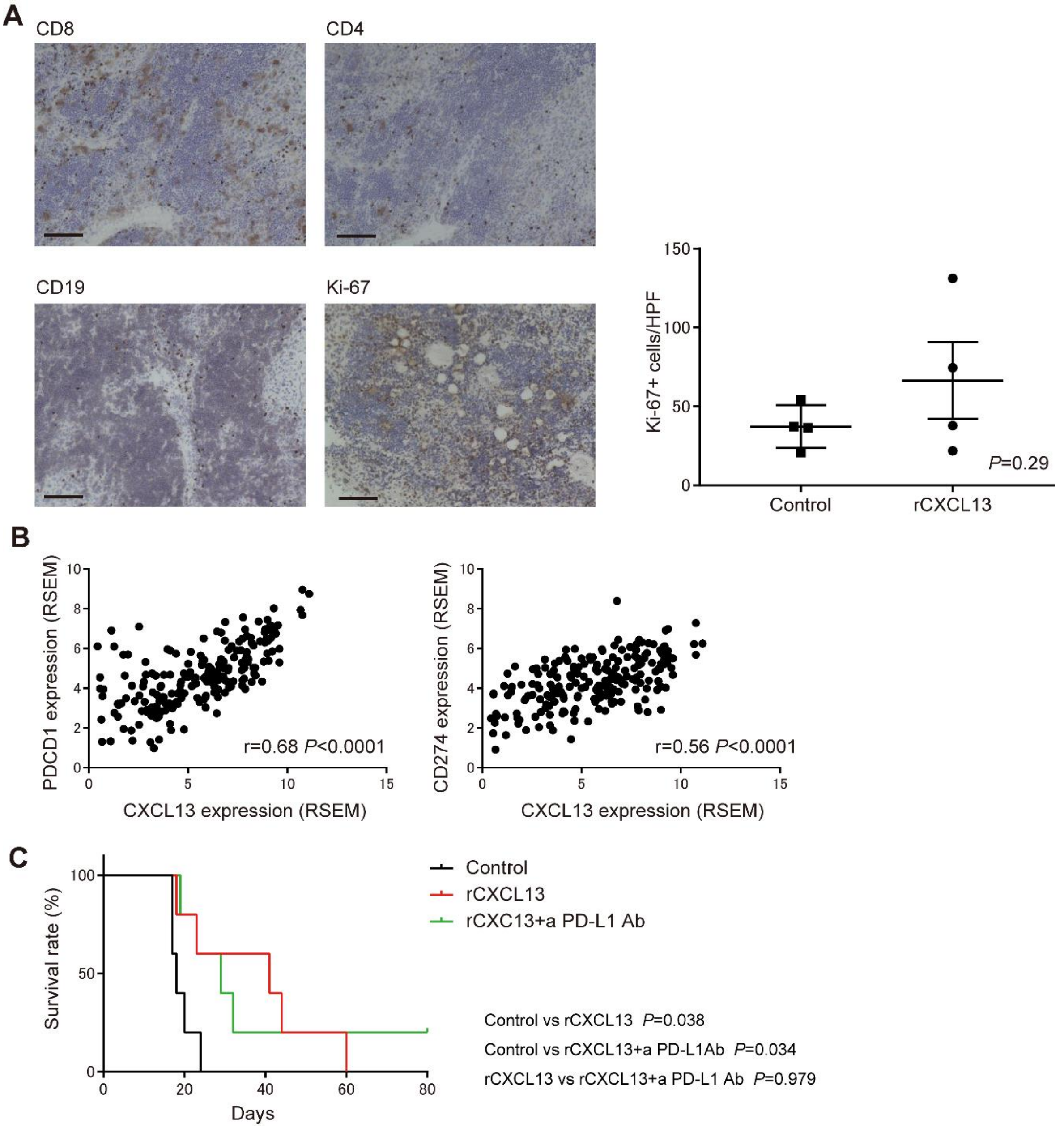

Supplemental Figure 5. Anti-tumor immunological effects of CXCL13 in a mouse ovarian cancer model

(A) Analysis of cells in the TLS induced by rCXCL13 in mice by IHC of CD8, CD4, CD19, and Ki-67 (DAB). Scale bars indicate 100 μm. The number of Ki-67 positive cells (mean count of five sections at high power field) in TLS was compared between the rCXCL13 treated group and the control group. Data are shown as the mean ± SEM of four samples. Statistical significance was determined by two-tailed Student's *t*-test. **(B)** Correlation between CXCL13 and PD-1 (PDCD1) or PD-L1 (CD274) expression in TCGA (n=217). Correlation was determined by Spearman's correlation test. **(C)** Survival curves of tumor-bearing mice treated with rCXCL13 or rCXCL13 + anti-PD-L1 antibodies. Analyses were performed using Kaplan-Meier estimates and log-rank tests. a PD-L1 Ab indicates anti PD-L1 antibody in (C).
